# Expression profile of epithelial-mesenchymal transition-related genes as a prognostic biomarker for endometrial cancer

**DOI:** 10.7150/jca.62729

**Published:** 2021-09-03

**Authors:** Lei Ye, Xiaojun Wang, Bilan Li

**Affiliations:** Department of Gynecology, Shanghai First Maternity and Infant Hospital, School of Medicine, Tongji University, Shanghai 200092, China.

**Keywords:** endometrial cancer, EMT, prognosis, Cox analysis, The Cancer Genome Atlas, survival

## Abstract

Epithelial-mesenchymal transition (EMT) is regulated by inducible factors, transcription factors, and a series of genes involved in diverse signaling pathways, which are correlated with tumor invasion and progression. In the present study, we analyzed the expression profile data of 1169 EMT-related genes in endometrial cancer (EC) from the Cancer Genome Atlas (TCGA) dataset, and performed consistency clustering to divide EC samples into two subgroups based on overall survival. The genes differentially expressed between the two subtypes included EMT-related genes. Univariate Cox analysis and least absolute shrinkage and selection operator (LASSO) were applied to construct a prognostic model based on the 44 genes signature. Five genes (L1CAM, PRKCI, ESR1, CDKN2A, and VIM) were finally included to establish a formula for prognostic risk score. The low-risk group showed significantly better prognosis compared with the high-risk group in the TCGA dataset. In addition, the risk-scoring model successfully predicted prognosis in an external GEO dataset (GSE102073). The relationship between ERα and vimentin levels was confirmed through immunohistochemistry. In conclusion, these data indicate that the expression profile of EMT-related genes could predict prognosis in EC.

## Introduction

Endometrial cancer represents the sixth commonest malignancy affecting women around the world, with 382,096 new diagnoses in 2018 1. The majority of endometrial cancer cases can be diagnosed and treated early, e.g. by surgical, radiation, hormonal and/or adjuvant therapies 2. However, those detected in advanced stage or recurrent and metastatic endometrial cancers respond poorly to chemotherapy and endocrine therapy. In this case, median survival is only 12 months 3, 4. Overall, 89,929 women died from endometrial cancer in 2018 1. Therefore, it is crucial to identify tools for early detection of endometrial cancer.

The classical endometrial cancer typing theory, which is based on the dualistic model of estrogen nuclear receptor expression, has a limited value in patient prognosis and treatment guidance. This suggests that clinical features and biology in this cancer are highly heterogeneous, which explain the off-target effects of molecular target tumor therapy, drug resistance, and the low objective response rate of immunotherapy 5, 6. Interestingly, the molecular typing theory could compensate for the above limitations, grouping endometrial cancer cases into the polymerase-epsilon (POLE) ultramutated, microsatellite instability hypermutated (MSI), copy-number low, and copy-number high categories 7. However, other common gene mutations of endometrial cancer besides POLE hypermutation cannot be categorized by the above system. For example, the most common *PTEN* (phosphate and tension homology deleted on chromosome ten) and* PIK3CA* (p110α catalytic subunit of PI3 kinase) gene mutations are found in all the above four types of endometrial cancer 8. These findings suggested that the molecular characteristics of tumor cells are not sufficient for endometrial cancer typing. Moreover, deepening the theoretical knowledge about endometrial cancer classification, and providing a breakthrough for clinical intervention need further investigation.

Epithelial cells undergo the epithelial-to-mesenchymal transition (EMT) process during tumor development, exhibiting the characteristics of mesenchymal cells and acquiring motility, invasion, and anti-apoptosis abilities 9. The reprogramming of gene expression during EMT and non-transcriptional changes are initiated and controlled by signalling pathways that respond to extracellular cues. Among these, TGF-β family signalling has a predominant role; however, the convergence of signalling pathways is essential for EMT 10. Previous studies demonstrated that EMT-related signaling pathways in endometrial cancer include the Wnt 11, transforming growth factor TGF-β 12, Hedgehog 13 and Notch 14 pathways. Interestingly, a study revealed that the EMT status has significant associations with peritoneal metastasis, progression-free survival and overall survival in ovarian cancer 16. In addition, recent reports have reported that EMT-related genes could serve as prognostic markers of neuroblastoma 17, ependymomas 18 and bladder cancer 19. However, in endometrial cancer, a comprehensive analysis of EMT-related genes for their prognostic value has not been reported.

Therefore, this study queried the Cancer Genome Atlas (TCGA) database to identify key genes responsible for the development of endometrial cancer, performing a comprehensive analysis of the associations of EMT-related genes with endometrial cancer development. In addition, consistency cluster, univariate Cox and LASSO analyses were carried out to construct a prognostic model, which was applied to distinguish high-risk from low-risk endometrial cancers. We found that the expression profile of EMT-related genes could predict EC patient prognosis.

## Materials and methods

### Data collection

The mRNA expression profile and clinical data of 543 primary endometrial cancer specimens were downloaded from https://portal.gdc.cancer.gov/. The gene mutation information of these samples was downloaded with the R package TCGA biolinks. Microsatellite instability scores were derived from a previous report 20. EMT-related genes were downloaded from the dbEMT database (http://dbemt.bioinfo-minzhao.org/).

The endometrial cancer samples used for model verification were from the GEO (https://www.ncbi.nlm.nih.gov/geo/) GSE102073 dataset, which included 85 tumor samples (not contained in the TCGA database); specimens with uncertain survival time were excluded from the subsequent analysis.

### Methods

#### Consistency clustering

The data of 1184 EMT-related genes were downloaded from the dbEMT database, including 1169 genes that were detected in TCGA endometrial cancer samples. The clinical information of the TCGA and GEO samples is summarized in Supplementary Tables 1 and 2. Next, a consistency clustering analysis of EMT gene expression profile in 543 endometrial cancer cases was performed, and the R package Consensus ClusterPlus was used for subsequent evaluation by Pearson's correlation (50 iterations and 80% resampling rate).

#### Differential gene expression and pathway enrichment analyses

The R package Limma was used to screen differentially expressed genes in both types of samples obtained by consistency clustering: fold change (FC) >2 (upregulation or downregulation); false discovery rate (FDR)-corrected P<0.05. The differentially expressed genes screened with the R package clusterProfiler underwent Gene Ontology (GO) and Kyoto Encyclopedia of Genes and Genomes (KEGG) pathway enrichment analyses.

#### Survival analysis

Survival analysis was performed by the Kaplan-Meier method and the log-rank test, based on multiple potential influencing factors.

#### Establishment and verification of the prognostic model

First, differentially expressed EMT-related genes were identified, and univariate Cox regression analysis was performed to determine those related to prognosis. Then, the LASSO method was applied to screen key prognosis-related genes and construct a prognostic model. The R package Maxstat was used to determine the optimal threshold for distinguishing patients with low-risk endometrial cancer from the high-risk group. Kaplan-Meier survival analysis was carried out to assess the predictive ability of the prognostic model, and the R package pROC 21 was used to generate receiver operating characteristic (ROC) curves.

#### Human tissue specimens

A total of 323 endometrial cancer samples were collected from Shanghai First Maternity and Infant Hospital from 2017 to 2019. None of the patients had received chemotherapy, immunotherapy, or radiotherapy before specimen collection. Histological classification and clinical staging were performed in accordance with the International Federation of Gynecology and Obstetrics classification system. The Ethics Committee of Shanghai First Maternity and Infant Hospital (Shanghai, China) approved this study, and the patients provided informed consent prior to specimen collection.

#### Immunohistochemistry (IHC)

IHC was performed as described previously 15, and the patients' information was showed in Supplementary Table 3. Specimens were confirmed by hematoxylin and eosin-stained sections. Formalin-fixed, paraffin-embedded sections (4 μm) were deparaffinized in xylene, rehydrated in graded alcohol, and rinsed in phosphate-buffered saline (PBS). Endogenous peroxidase activity was blocked with 3% hydrogen peroxide in methanol for 20 min. Epitope retrieval was performed in citrate buffer for 5 min at 100 °C. Slides were incubated with antibodies at 4 °C overnight. After washing three times with fresh PBS, the sections were subsequently incubated with secondary antibody (Cell Signaling Technology) at room temperature (37 °C) for 30 min. For visualization of the reaction, the diaminobenzidine-tetrahydrochloride was stained, then counterstained with hematoxylin, dehydrated and cover slipped. Vimentin and estrogen receptor (ER) alpha levels were classified into three categories: negative, weakly positive, and strongly positive. Two pathologists independently evaluated the specimens in a blinded manner. Anti-vimentin (1:200, #ab92547) and anti-ERα (1:250, #ab108398) primary antibodies (Abcam, UK) were employed.

#### Statistical analysis

One-way analysis of variance (ANOVA) was performed to analyze the associations of EMT-related genes with clinical characteristics (stage and grade). The t-test was carried out to assess differences in microsatellite instability, *PTEN* mutation, and tumor suppressor p53 (*TP53*) mutation in endometrial cancer. In addition, univariate and multivariate Cox regression analyses were performed to determine risk scores and the prognosis values of various clinical and molecular characteristics. The log-rank test and Kaplan-Meier method were applied to evaluate overall survival (OS) in the high- and low-risk groups, and samples with different grades or stages. All statistical analyses were conducted with R version 3.6.2.

## Results

### Expression patterns of EMT-related genes and pathological features in endometrial cancer

**Figure 1:**
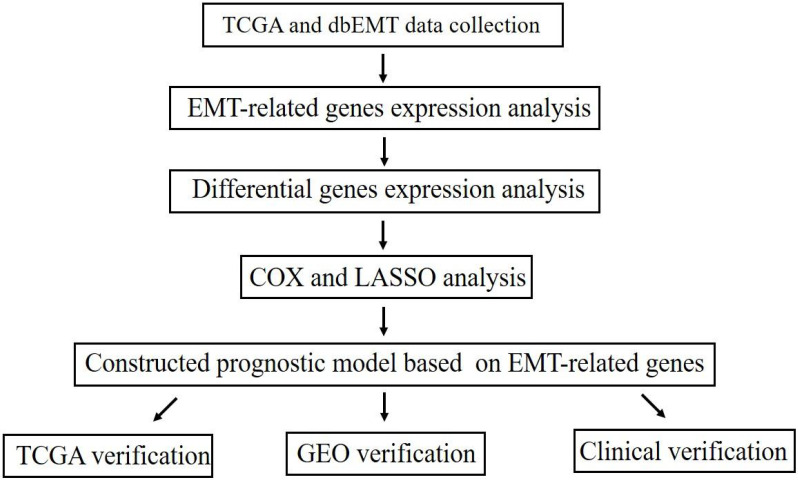
Study flowchart.

**Figure 2:**
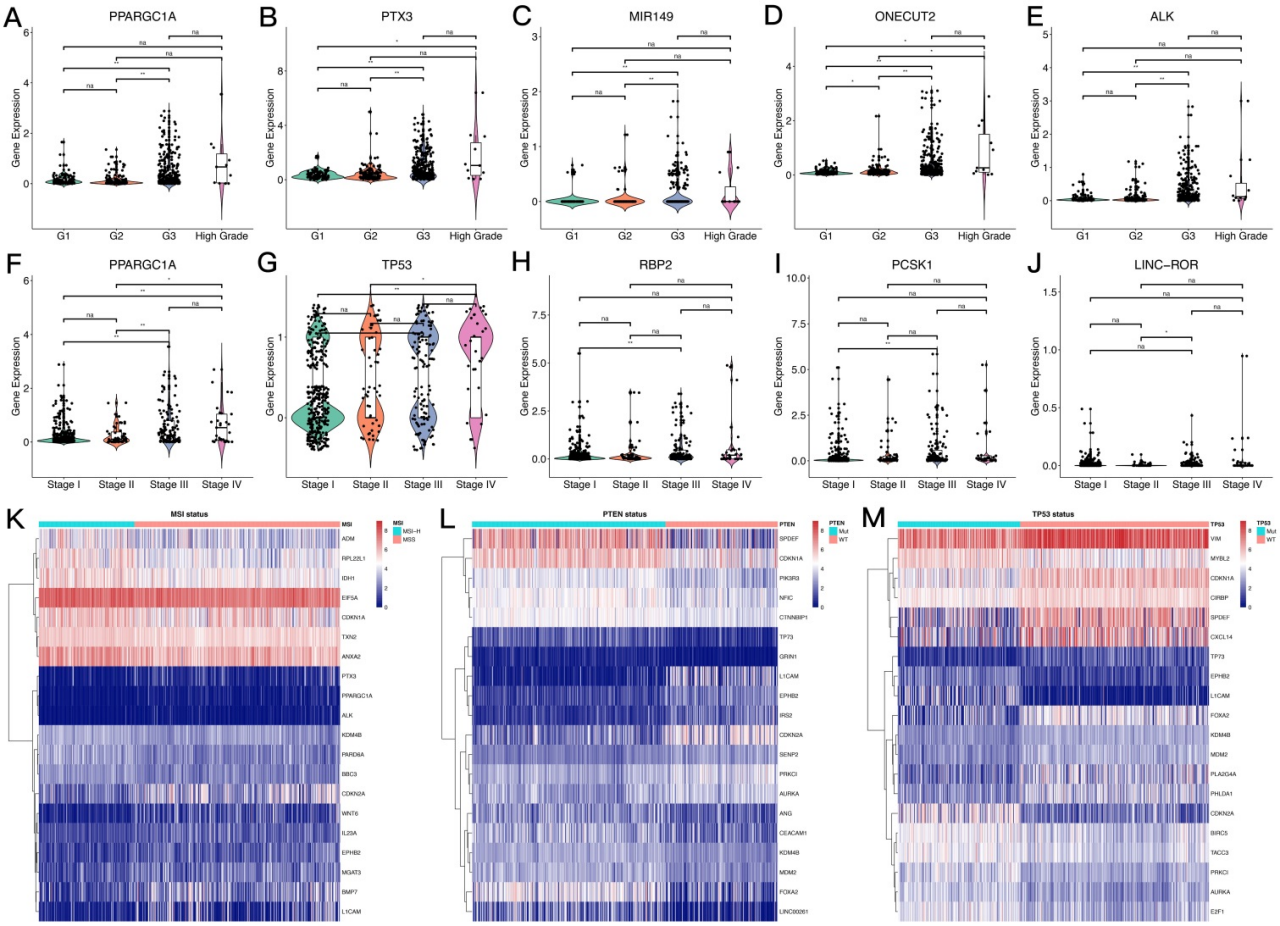
** Expression levels of EMT-related genes in endometrial carcinoma cases with different clinicopathological features. A-J.** Box scatter plots of gene expression distributions. ns, P>0.05; *P<0.05; ** P<0.01; ***P<0.001; ****P<0.0001. **K-M.** Heat maps of the top 20 EMT-related genes in samples from different subgroups.

The overall process for selecting and analyzing EMT-related genes in endometrial cancer is shown in Figure 1. Since EMT is considered to be closely related to tumors, the associations of clinical characteristics, molecular characteristics, and gene expression in endometrial cancer were systematically investigated in TCGA, including tumor grade and stage, microsatellite instability, and *TP53* and *PTEN* gene mutations. The expression level distributions of select EMT-related genes in different clinical characteristics (grade and stage) are shown in Figure 2A-J. According to microsatellite instability status and *TP53* and *PTEN* gene mutations, the samples were divided into the microsatellite instability (MSI)/microsatellite stability (MSS), *TP53* mutation/*TP53* wild type, and *PTEN* mutation/*PTEN* wild type. Then, the top 20 EMT-related genes with differential expression between the two sample groups were screened for heat map generation (Figure 2K-M).

### Consistency clustering of EMT-related genes categorizes samples into two subgroups with significantly different prognoses

**Figure 3:**
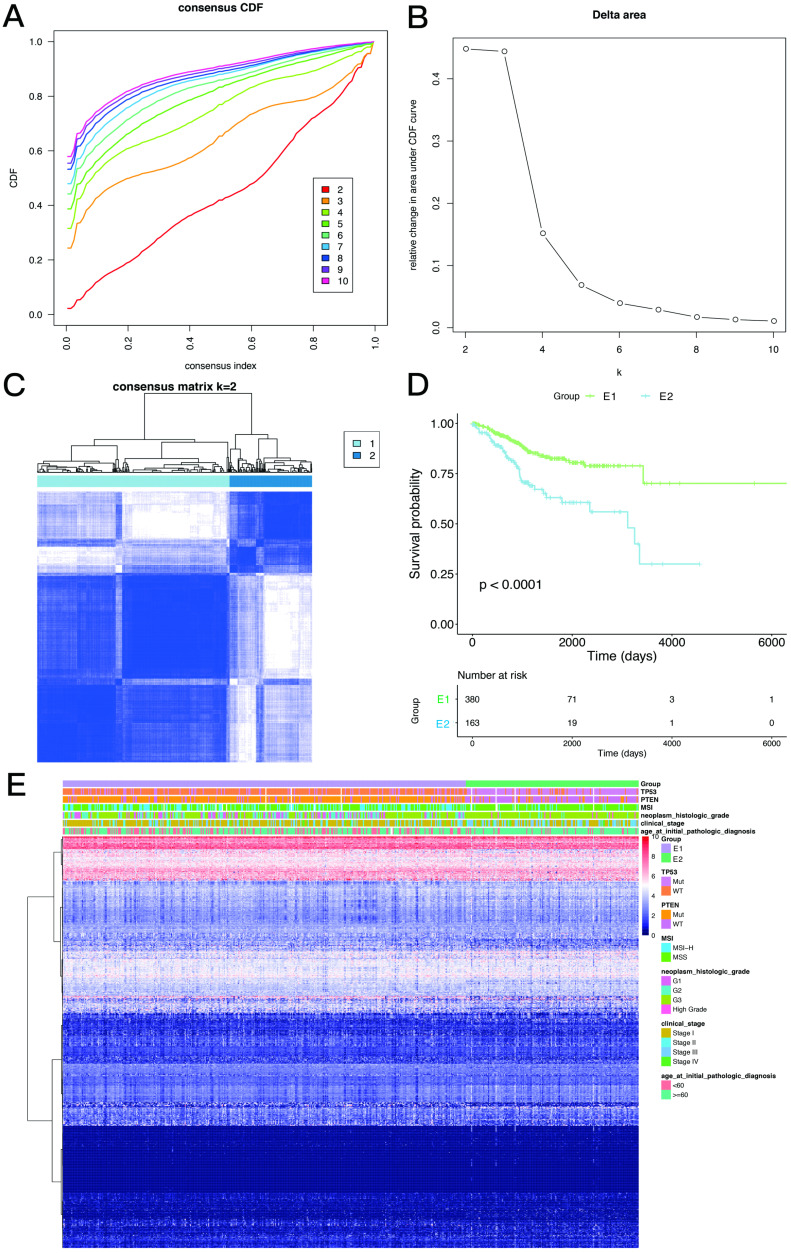
** Consistency clustering of EMT-related genes. A,B.** Clustering cumulative distribution function and relative change in area under the Cumulative Distribution Function (CDF) curve. **C.** Correlation between the EMT-1 (E1) and EMT-2 (E2) subgroups. **D.** Kaplan-Meier survival curves for both groups. **E.** Heat maps depicting the expression levels of EMT-related genes in both sample groups.

Based on the expression similarity of EMT-related genes, k=2 was selected for consensus matrix building (Figure 3A,B). The 543 samples from endometrial cases in the TCGA dataset were divided into the EMT-1 (E1) and EMT 2 (E1) subgroups, encompassing 380 and 163 samples, respectively. The E1 subgroup showed longer OS compared with the E2 subgroup (Figure 3C,D), indicating that these two clusters might be closely related to the occurrence and development of endometrial cancer. The expression heat maps of EMT-related genes in both sample groups are shown in Figure 3E. Together, these data suggest that the expression patterns of EMT-related genes was closely related with EC prognosis.

### Differences in sample groups obtained by consistency clustering

**Figure 4:**
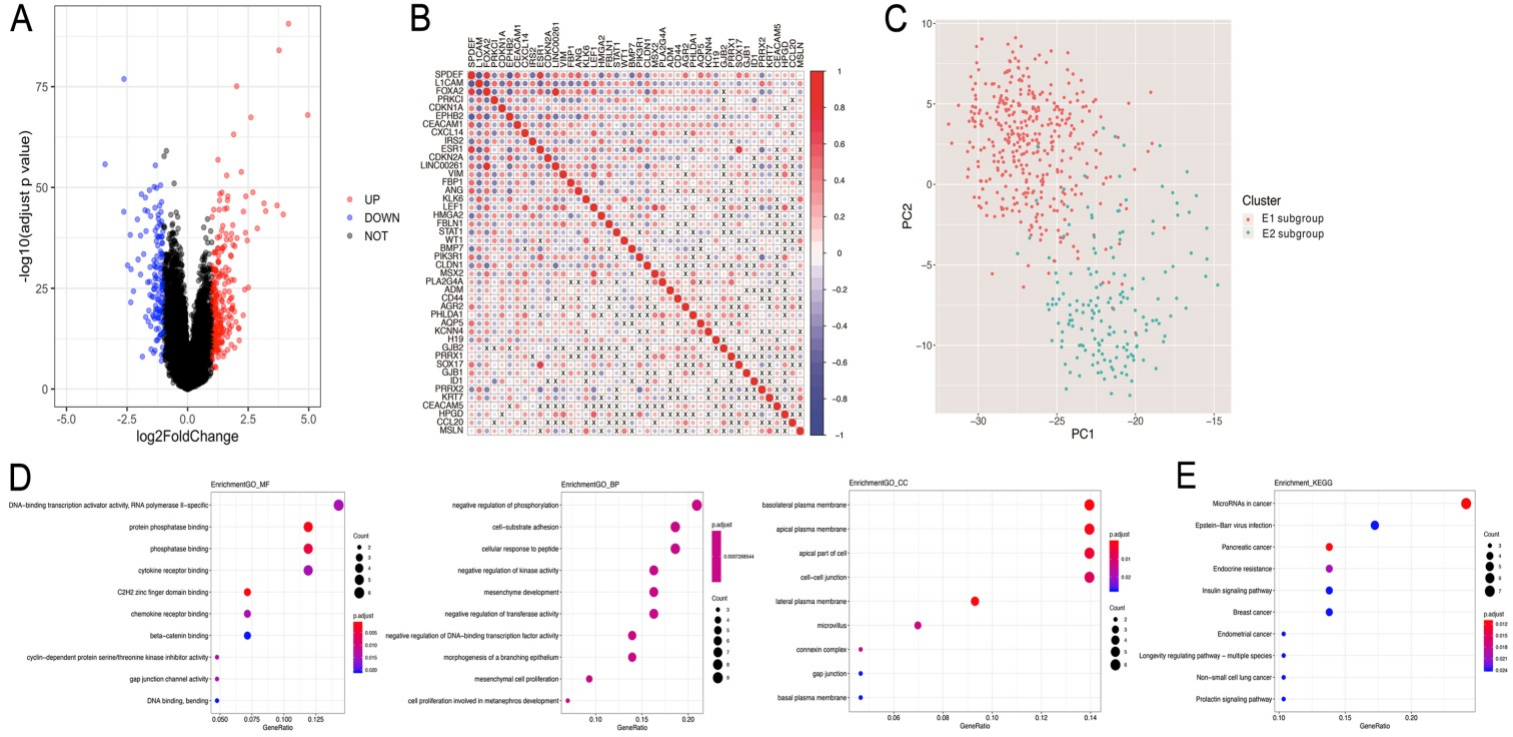
** Differentially expressed genes and pathway enrichment analysis. A.** Volcano plot generated with the R package Limma, depicting the expression levels of genes obtained by consistency clustering. **B.** Pearson's correlation analysis of the expression levels of EMT-related differential genes (x, non-significant correlation at P>0.05). **C.** Principal component analysis (PCA) of differentially expressed genes related to EMT. **D.** Pathway enrichment analysis. Gene Ontology analysis suggested that Molecular Function (GO-MF), Gene Ontology analysis suggested that Biology Process (GO-BP), Gene Ontology analysis suggested that Cellular Component (GO-CC) and KEGG analysis were performed.

Subsequently, the differential gene analysis between the E1 and E2 groups was analyzed by Limma. As shown in Fig. 4A, among the 370 differentially expressed genes, 237 were upregulated, while 133 were downregulated. Interestingly, 44/370 genes were related to EMT.

In order to assess the interactions among these 44 EMT-related differential genes, we performed correlation analyses, and found significant associations among multiple EMT genes (Figure 4B). Then, principal component analysis (PCA) was performed to compare the transcription profiles of the E1 and E2 subgroups, and different types of samples with fewer parameters were distinguished using dimension reduction for multidimensional data. The results showed a significant difference between E1 and E2, indicating that 44 EMT genes could be used to distinguish the two groups of samples (Figure 4C).

Subsequently, the identified EMT-related genes with differential expression between the two subgroups were subjected to pathway enrichment analysis based on the GO and KEGG pathways (Figure 4D,E). The results showed that the 44 differentially expressed EMT-related genes were mainly involved in related functions and biological processes, including negatively regulated phosphorylation, cell-matrix adhesion, Epstein-Barr virus infection, and endometrial cancer.

### Identification of EMT-related differential genes associated with OS

**Figure 5:**
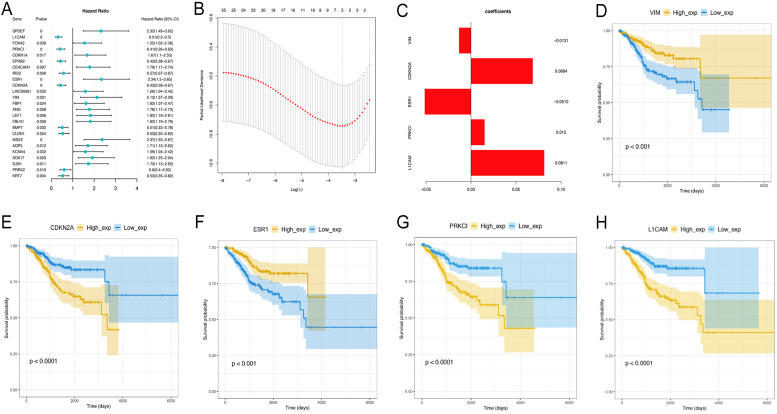
** Identification of EMT-related differential genes most associated with overall survival. A-C.** The LASSO analysis is to select variables for the model from the 25 candidates, which screen five key prognosis-related genes, whose expression levels and LASSO regression coefficients were employed to establish a risk-scoring model for predicting patient survival. **D.** Survival analyses based on low versus high L1CAM and PRKCI amounts.

Univariate Cox analysis was performed to assess the above 44 differentially expressed EMT-related genes, and 25 were significantly correlated with prognosis (P<0.05) (data not shown). Next, the LASSO method (Figure 5A-C) was employed to screen five key prognosis-related genes , whose expression levels and LASSO regression coefficients were employed to establish a risk-scoring model for predicting patient survival as follows: risk score = 0.08112529 × L1CAM levels + 0.01497074 × PRKCI levels - 0.05119486 × ESR1 levels + 0.06843238 × CDKN2A levels -0.01311061 × vimentin levels. Survival analyses based on low versus high L1CAM and PRKCI amounts are shown in Figure 5D-H. Together, these data suggest that the risk-scoring model may be applied in prediction of EC patients' survival.

### Application of the model in the TCGA and GSE102073 datasets

**Figure 6:**
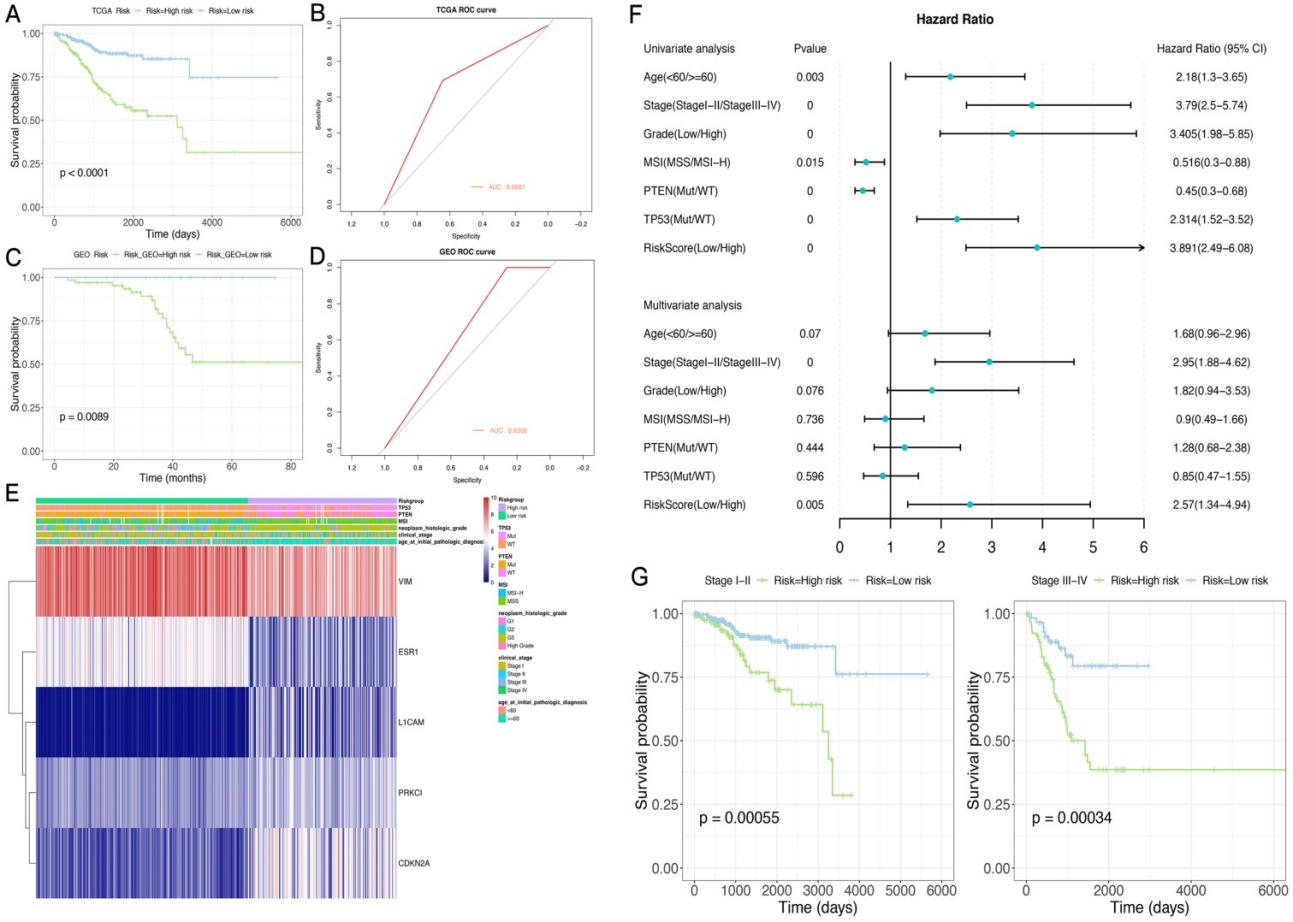
** Application and performance of the novel EMT-gene based model. A.** Survival curves in both sample groups in the TCGA data set. **B.** ROC curve analysis of the risk score in the TCGA data set. **C.** Survival curves in both sample groups in the GEO data set. **D.** ROC curve analysis of the risk score in the GEO data set. **E.** Heat maps of gene expression in both sample groups in the TCGA set. **F.** The risk score and other clinical and molecular features are significantly correlated with OS. **G.** ROC curve analysis of tumor stage (stage I-II or stage III-IV) in predicting OS.

A risk score was determined for each patient in the TCGA dataset, and the optimal cutoff value of -0.005079712 was obtained using the R package Maxstat to classify the patients into the high-risk and low-risk groups. Prognosis was better in the low-risk group compared with the high-risk group. ROC curve analysis indicated that the risk score could predict patient survival with an area under the ROC curve (AUC) of 0.6681 (Figure 6A,B). The heat maps of gene expression in both sample groups in the model are shown in Figure 6E.

Subsequently, 84 endometrial cancer samples in the GSE102073 dataset were employed for model validation. The optimal threshold calculated with the R package Maxstat (-0.08225368) was utilized to separate the high- and low-risk groups. As shown in Figure 6C,D, prognosis was significantly worse in the high-risk group compared with the low-risk group. An AUC of 0.6308 was determined in this dataset for the model.

### The novel risk score has good prognostic performance, and is related to the clinical characteristics of the sample population

Next, the risk-scoring model was assigned to each clinicopathological feature in the TCGA dataset. Significant differences were found between the high- and low-risk groups in terms of age, tumor stage and grade, and MSI status (Supplementary Figure 1). The ROC curves showed that the risk score could accurately predict the survival rate (AUC=0.69), the MSI status (AUC=0.68), the PTEN mutation status (AUC=0.85), and the TP53 mutation status (AUC=0.86) in patients with endometrial cancer. In addition, the risk score had higher prediction accuracy compared with tumor stage and grade.

In order to test whether this prognostic model is an independent prognostic factor of endometrial cancer, univariate Cox analysis in the TCGA dataset was performed. As shown in Figure 6F,G, the model and other clinical and molecular features were significantly related to OS. Then, clinicopathological variables were adjusted before entering in multivariate Cox analysis, which showed that the risk score and stage (stage I-II or stage III-IV) were significantly correlated with OS (Figure 6F,G). Indeed, prognosis differed significantly between the high- and low-risk groups as well as stage I-II and III-IV subgroups.

The CIBERSORT method was combined with the LM22 characteristic matrix to further estimate the difference in the number of immune infiltrating cells among the 22 immune cell types in the TCGA high- and low-risk subgroups. Within and between the groups, the proportion of immune cells varied among the samples and was analyzed by T-test. The results showed that the proportions of immune cells, such as Tregs cells, activated dendritic cells, and macrophages M1, were significantly different in the two groups of samples (Supplementary Figure 2).

### Clinical validation of two differentially expressed EMT-related genes identified in the risk scoring model by IHC

**Figure 7:**
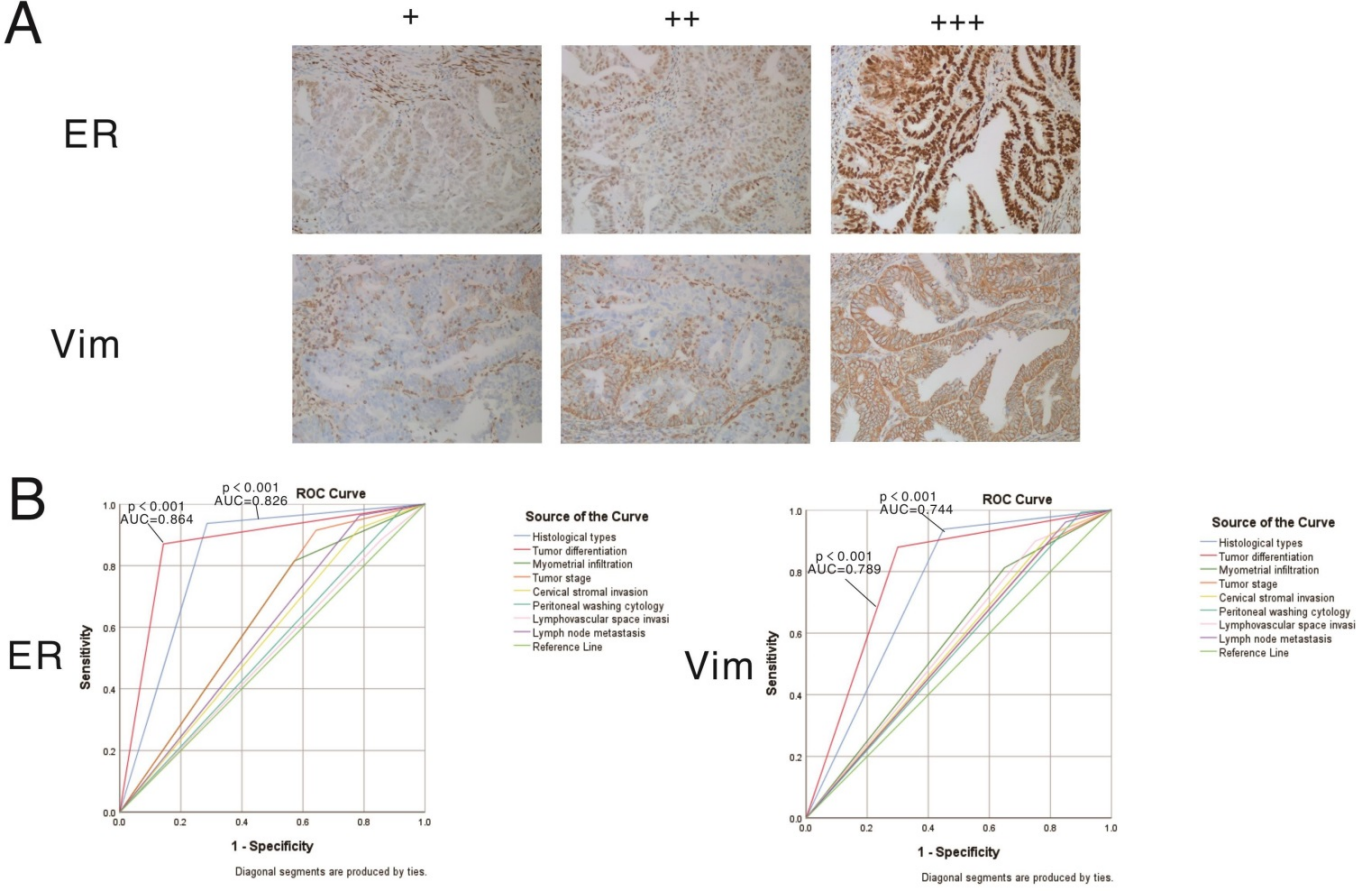
** Expression patterns and prognostic values of vimentin and ERα in endometrial cancer samples. A.** Representative immunohistochemistry (IHC) micrographs are shown for both proteins (magnification, 200×). **B.** Performance analysis of vimentin and ERα expression levels in predicting prognosis in endometrial cancer.

The expression patterns of vimentin and ERα in endometrial cancer samples were examined by IHC (Figure 7). Positive immunostaining signals of both vimentin and ERα were significantly higher in tumors with higher differentiation compared with lowly differentiated samples, and significantly correlated with early stage (stage I/II) disease rather than advanced stage (stage III/IV) cases (all P<0.001). No staining of ERα was significantly correlated with deeper invasion of myometrial infiltration, cervical stromal invasion, and lymph node metastasis. On the other hand, no staining of vimentin was significantly correlated with endometrioid cancer, deeper invasion of myometrial infiltration, peritoneal washing cytology positive, cervical stromal invasion, and lymph node metastasis (showed in Tables 3 & 4).

## Discussion

In this study, the expression profile of EMT-related genes could predict prognosis in EC, and five genes were used to establish a formula for prognostic risk score calculation. The risk scoring model was effective in distinguishing high-risk from low-risk cases, and these data were validated in the GSE102073 dataset.

A recent study reproduced Asian Cancer Research Group (ACRG) gastric cancer (GC) subtypes using different platforms, and predicted EMT subtypes based on 10 genes; a follow up prospective clinical study will exploit these data for precision oncology in GC 22. This study systematically analyzed the associations of EMT related genes in endometrial cancer with different clinicopathological features. As shown above, the expression patterns of EMT-associated genes were closely correlated with malignant clinicopathological features in endometrial cancer. Five most significant genes were selected, including L1CAM (L1 cell adhesion molecule), PRKCI, ESR1, CDKN2A and VIM, to construct a prognostic model for endometrial cancer.

In addition to factors required for EMT in the TGF-β pathway in the novel prognostic model, L1CAM is highly critical for the progression of human tumors^23^. Indeed, downstream signaling and regulation of L1CAM during tumor progression and EMT dually act as cell adhesion and/or motility promoting events^24^. Schafer et al. found that TGF-β1-mediated upregulation of L1CAM triggers the binding of integrins, resulting in enhanced overlapping TGF-β1-integrin signaling, which finally leads to NF-κB activation. L1CAM expression can induce IL-1β secretion and NF-κB activation not only in tumor cells but also in immune cells. This could promote a pro-tumorigenic environment and EMT induction, leading to aggressive and invasive tumor growth 25.

Two additional EMT-related genes were included in the prognostic model, including CDKN2A and PRKCI. The former is positively regulated in hepatocellular carcinoma (HCC) 26. Meanwhile, PRKCI gene expression in cancerous tissue might be a useful prognostic factor in gastric cancer after gastrectomy 27. In addition, PRKCI upregulation is an early and common event in ovarian neoplasms and promotes an immune-suppressive tumor microenvironment in tumor progression 28. The mechanisms by which these 2 genes affect the EMT process in endometrial cancer should be further explored. In this study, L1CAM, PRKCI and CDKN2A were the most relevant genes affecting survival in endometrial cancer, with high amounts reflecting poor prognosis.

Vimentin, a crucial effector in EMT progression, controls TGF-β1-Slug signaling and EMT processing. In pancreatic ductal adenocarcinoma (PDAC), cell-surface vimentin is a biomarker for isolating CTCs 29-32. Estrogen receptor-α (ERα) status in endometrial carcinoma, associated with poor prognosis, is reflected in transcriptional signatures suggesting targets for novel therapeutics 33. Specifically, lack of ER-α in endometrial cancer is associated with EMT and reduced survival. Wik et al. reported that ERα could predict the response to PI3K/mTOR inhibitors in clinical trials and suggest EMT inhibitors for ERα-negative endometrial cancer 13, 34, 35. Tu et al. found that TGF-β1-mediated EMT is associated with ER. It was shown that decreased amounts of vimentin and snail result in reduced ER expression by small interfering RNA-mediated silencing, thereby preventing the TGF-β-induced EMT 36. In the above clinical validation, vimentin and ERα could act as biomarkers to distinguish the high risk factors of endometrial cancer.

In this study, the high- and low-risk groups based on the prognostic model were closely associated with the P53 and PTEN mutation statuses. TP53 is well-known as one of the most frequently mutated genes in various cancer types. For instance, TP53 mutants occur in more than 60% of colorectal cancer (CRC) cases. Although TP53 mutations are highly recurrent in serous endometrial carcinomas (SECs), they are also present in a subset of endometrioid endometrial carcinomas (EECs) 37, 38. Frequent mutations were found in TP53, PTEN, PIK3CA, PPP2R1A, FBXW7, and KRAS, similar to endometrioid and serous uterine carcinomas. Transcriptome analysis identified a strong EMT gene signature in a subset of cases attributable to epigenetic alterations 39, 40. This study revealed that EMT genes are correlated with multiple cellular processes, e.g., cell adhesion, cell-cell signaling and TGF-β signaling pathways, including cell cycle, p53 signaling and PTEN signaling. Further work is needed to determine the functional aspects of these associations, which will be important to verify our conclusion.

The interaction between immune microenvironment and cancer cells is important for tumor progression 41. Our results also reveal that EMT genes are related with immune microenvironment and immune cell infiltration. A series of studies have explored the prognostic and therapeutic value of programmed death-1(PD-1)/ programmed death-ligand 1 (PD-L1) in patients with endometrial cancer (EC); however, the effect of them are controversial, such as Pembrolizumab, an antibody targeting PD-1, which just has moderate efficacy 42. Funaki et al. investigated the mechanism of PD-L1 expression as well as changes in its expression during the EMT process in non-small cell lung cancer (NSCLC). Hsu et al. identified a non-canonical MET activity of etoposide, which suppresses the EMT/β-catenin/PD-L1 axis through TOP2B degradation-dependent nuclear β-catenin reduction, leading to PD-L1 downregulation of CSCs and non-CSCs and sensitization of cancer cells to anti-Tim-3 therapy 43. In PDAC, the expression of total Vimentin protein was positively correlated with PD-L1 and inhibited CD8+ T-cell activation in patients with GC. Notably, co-expression of PD-L1 and cell-surface VIM (CSV) was detected in locally advanced GC tumor specimens and metastatic lymph nodes. Therefore, the potential of molecularly targeted immunotherapy to treat those EMT-related transcription factors could be a potential strategy to enhance cancer immunotherapy efficacy.

Although we have identified and validated that five genes established a formula for prognostic risk score calculation to identify the lower risk and higher risk groups in EC patients, this study has some limitations. First, only data from TCGA data base was utilized. Therefore, more independent data sets are needed for further verification. Secondly, further studies are needed to clarify their mechanisms in the tumorigenesis and progression of EC. Thirdly, more biological experiments are needed to verify and explore the mechanism of those five genes. We are currently conducting clinical trials, but this is a very time-consuming process. We will verify our conclusion in the follow-up study.

In conclusion, the approach developed in this study aimed to retype endometrial cancer based on characteristic genes related to EMT in the tumor microenvironment. The samples were divided into two groups based on significantly different prognoses, as assessed by consistency clustering of EMT gene expression profiles. Then, univariate Cox regression and LASSO analyses were performed to establish a risk model including differentially expressed genes related to EMT. The novel model could predict the survival of endometrial cancer patients, with the derived risk score showing a satisfactory prognostic performance. These findings suggest that the expression profile of EMT-related genes could predict prognosis in EC, which could help design proper treatment plans in these patients.

## Supplementary Material

Supplementary table 1.

Supplementary table 2.

Supplementary table 3.

## Figures and Tables

**Table 1:** TCGA Sample Information

Clinical Features	Number
**Age**	
≥60	363
<60	178
NA	2
**Stage**	
Stage I	337
Stage II	51
Stage III	126
Stage IV	29
**Grade**	
G1	98
G2	119
G3	315
High Grade	11
**Status**	
Alive	452
Dead	91

**Table 2:** GEO Sample Information

Clinical Features	Number
**Age**	
≥60	36
<60	48
**Stage**	
Stage I	1
Stage II	3
Stage III	55
Stage IV	25
**Status**	
Alive	65
Dead	19

**Table 3:** Association between the expression of Vimentin and clinico-pathological features in patients with endometrial cancer (n=311)

			Positive	Negative	Contingency Coefficient	p Value
**Age at initial pathologic diagnosis**	<60	Count	188	9	0.165	0.003
		percent	95.4%	4.6%		
	≥60	Count	98	16		
		percent	86.0%	14.0%		
**Pre-operative CA125**	<35	Count	236	18	0.055	0.337
		percent	92.9%	7.1%		
	≥35	Count	49	6		
		percent	89.1%	10.9%		
**Histological types**	G1-G3	Count	265	12	.363	0.000
		percent	95.7%	4.3%		
	Non-endometriod cancer	Count	21	13		
		percent	61.8%	38.2%		
**Tumor differentiation**	Low	Count	36	16	0.353	0.000
		percent	69.2%	30.8%		
	Medium	Count	28	0		
		percent	100.0%	0.0%		
	High	Count	222	9		
		percent	96.1%	3.9%		
**Myometrial infiltration**	<0.5	Count	235	15	0.150	0.007
		percent	94.0%	6.0%		
	≥0.5	Count	51	10		
		percent	83.6%	16.4%		
**Tumor stage**	I-II	Count	256	19	0.114	0.043
		percent	93.1%	6.9%		
	III-IV	Count	30	6		
		percent	83.3%	16.7%		
**Cervical stromal invasion**	Negative	Count	262	21	0.072	0.202
		percent	92.6%	7.4%		
	Positive	Count	24	4		
		percent	85.7%	14.3%		
**Peritoneal washing cytology**	Negative	Count	254	18	0.196	0.001
		percent	93.4%	6.6%		
	Positive	Count	2	2		
		percent	50.0%	50.0%		
**Lymphovascular space invasion**	Negative	Count	257	18	0.150	0.007
		percent	93.5%	6.5%		
	Positive	Count	29	7		
		percent	80.6%	19.4%		
**Lymph node metastasis**	Negative	Count	275	20	0.195	0.000
		percent	93.2%	6.8%		
	Positive	Count	11	5		

**Table 4:** Association between the expression of ER and clinico-pathological features in patients with endometrial cancer (n=323)

			Positive	Negative	Contingency Coefficient	p Value
**Age at initial pathologic diagnosis**	<60	Count	197	9	0.053	0.340
		percent	95.6%	4.4%		
	≥60	Count	109	8		
		percent	93.2%	6.8%		
**Pre-operative CA125**	<35	Count	250	12	0.039	0.483
		percent	95.4%	4.6%		
	≥35	Count	55	4		
		percent	93.2%	6.8%		
**Histological types**	G1-G3	Count	284	4	0.446	0.000
		percent	98.6%	1.4%		
	Non-endometriod cancer	Count	22	13		
		percent	62.9%	37.1%		
**Tumor differentiation**	Low	Count	41	15	0.404	0.000
		percent	73.2%	26.8%		
	Medium	Count	29	0		
		percent	100.0%	0.0%		
	High	Count	236	2		
		percent	99.2%	0.8%		
**Myometrial infiltration**	<0.5	Count	251	9	0.162	0.003
		percent	96.5%	3.5%		
	≥0.5	Count	55	8		
		percent	87.3%	12.7%		
**Tumor stage**	I-II	Count	277	10	0.219	0.000
		percent	96.5%	3.5%		
	III-IV	Count	29	7		
		percent	80.6%	19.4%		
**Cervical stromal invasion**	Negative	Count	282	12	0.166	0.002
		percent	95.9%	4.1%		
	Positive	Count	24	5		
		percent	82.8%	17.2%		
**Peritoneal washing cytology**	Negative	Count	267	13	0.110	0.062
		percent	95.4%	4.6%		
	Positive	Count	3	1		
		percent	75.0%	25.0%		
**Lymphovascular space invasion**	Negative	Count	271	13	0.083	0.136
		percent	95.4%	4.6%		
	Positive	Count	35	4		
		percent	89.7%	10.3%		
**Lymph node metastasis**	Negative	Count	293	13	0.189	0.001
		percent	95.8%	4.2%		
	Positive	Count	13	4		
		percent	76.5%	23.5%		
